# Neural mechanisms underlying the effects of visual input on static balance in older adults with mild cognitive impairment

**DOI:** 10.3389/fnins.2026.1745957

**Published:** 2026-03-27

**Authors:** Hongen Liu, Weihua He, Jiajie Zhu, Yan Chen, Xin Yang, Yongbo Wang, Yanbai Han, Yiming Han

**Affiliations:** 1 Guangxi Normal University, Guilin, China; 2 Jinan Third People's Hospital, Jinan, China; 3 Shandong Sport University, Jinan, China

**Keywords:** balance control, brain network, fNIRS, mild cognitive impairment, visual input

## Abstract

**Objective:**

This study aimed to examine the role of visual information in static balance control and to explore the coupling between brain network topological properties and balance performance in older adults with mild cognitive impairment (MCI).

**Methods:**

Thirty-four older adults with MCI and 34 cognitively normal (CN) performed two-leg standing tasks with eyes open and eyes closed on a three-dimensional force platform to assess center of pressure indices. fNIRS was used to evaluate the topological properties of brain networks across regions of interest.

**Results:**

Compared with the eyes-open condition, the MCI group showed significantly greater sway area (*p* < 0.001) and anteroposterior RMS (*p* = 0.049), along with significantly lower small-worldness (*p* < 0.001) and nodal efficiency in the right primary somatosensory cortex (R-S1) (*p* = 0.002) under the eyes-closed condition. Similarly, in the CN group, sway area (*p* = 0.018) was significantly larger and small-worldness (*p* = 0.001) was significantly lower under the eyes-closed condition. Moreover, under the eyes-closed condition, the MCI group exhibited a significantly larger sway area (*p* = 0.030) and lower small-worldness (*p* < 0.001) than the CN group. Correlation analyses revealed that sway area was negatively correlated with small-worldness in the MCI group (*r* = −0.384, *p* = 0.025), and anteroposterior RMS was negatively correlated with R-S1 nodal efficiency in the CN group (*r* = −0.427, *p* = 0.012).

**Conclusion:**

Older adults with MCI show reduced balance control under visual restriction. The association between impaired balance and decreased brain network efficiency suggests that disrupted neural organization contributes to balance deficits in MCI.

## 1 Introduction

Mild Cognitive Impairment (MCI) represents a transitional stage between normal aging and dementia, with the rate of progression to dementia reported to reach 60–100% within 5–10 years (Xue et al., 2018). Consequently, MCI is regarded as a major risk factor for Alzheimer’s disease. Previous studies have indicated that individuals with MCI exhibit potential impairments in postural and balance control. Compared with cognitively normal older adults, they show reduced gait stability and are more susceptible to imbalance and falls in daily activities. Moreover, as cognitive function declines, their risk of falling increases significantly (Iso-Markku et al., 2024). Falls among older adults can result not only in severe outcomes such as fractures and traumatic brain injury, but also in physical deterioration, long-term disability, and even death, thereby imposing a substantial burden on families and public health systems (Yang et al., 2020; Ismail et al., 2021; Sherman et al., 2021).

Balance control is a complex neuromuscular process that serves as one of the major risk factors for falls in older adults and forms the foundation for maintaining postural stability and performing daily activities (Zahedian-Nasab et al., 2021). Previous studies have shown that a decline in balance ability is closely associated with structural alterations in the brain and cognitive deterioration (Halvarsson et al., 2015). Moreover, effective balance control requires the continuous engagement of cognitive resources (Vallabhajosula et al., 2015). Cognitive decline can directly impair motor coordination and balance responses during postural maintenance (Basta et al., 2019; Park, 2024), thereby leading to poorer balance performance in standing tasks among older adults (Bhagwat and Deodhe, 2023; Numakawa and Odaka, 2022).

However, postural control depends not only on cognitive resources but also on the integration of multisensory inputs, including visual (Park, 2024), vestibular, and proprioceptive information (Xing et al., 2023), which are coordinated by the central nervous system to maintain body balance (Lelard et al., 2019; Sultan et al., 2021). Among these sensory modalities, the visual system plays a particularly critical role in maintaining postural stability. When visual input is limited, postural sway increases significantly (Hsu et al., 2007), and individuals with visual impairments rely more heavily on proprioceptive and vestibular cues to compensate (Friedrich et al., 2008). Aging further alters the sensory weighting of multisensory systems in postural control (Faraldo-García et al., 2012), diminishing older adults’ ability to adapt to external perturbations—a phenomenon that becomes especially evident when sensory cues are obstructed or unreliable (de Dieuleveult et al., 2017). This phenomenon is associated not only with neuromuscular decline but also with reductions in cognitive and visuospatial processing abilities (Takacs et al., 2013). Therefore, investigating the interactive mechanisms between cognitive function and multisensory integration in postural control among older adults is essential for understanding fall risk and for developing effective intervention strategies.

Although recent studies have begun to investigate neural connectivity and multisensory processing during postural control in aging, demonstrating age-related alterations in cortical network interactions under sensory conflict (Wang et al., 2024; Wang et al., 2025b), several important gaps remain. Most existing evidence has focused on healthy older adults or training populations, with limited attention to individuals with MCI. Moreover, prior work has predominantly examined directed or effective connectivity, whereas the large-scale topological organization of cortical functional networks assessed using graph-theoretical approaches remains insufficiently characterized in relation to standing balance. In particular, how cortical network topology in MCI responds to systematically manipulated visual conditions has not been systematically examined. Therefore, further investigation at the systems-network level is warranted to clarify how cognitive decline alters multisensory integration and balance regulation.

Given the upright standing paradigm employed in this study, an ecologically valid neuroimaging modality was required. Functional near-infrared spectroscopy (fNIRS) permits cortical measurement during natural standing and is relatively tolerant to motion compared with Functional Magnetic Resonance Imaging (fMRI). Although electroencephalography (EEG) offers superior temporal resolution, it is more vulnerable to motion and muscle artifacts during balance tasks. The HbO signal obtained from fNIRS provides stable indices for functional connectivity and graph-theoretical network analysis. Despite its lower temporal resolution and limited subcortical coverage, fNIRS aligns well with the present focus on large-scale cortical network topology under different visual conditions.

Accordingly, this study employed fNIRS combined with a bipedal standing balance task to systematically evaluate balance performance and brain network topological characteristics in older adults with MCI under different visual conditions. The study focused on analyzing the associations between standing balance and brain network metrics in older adults with MCI and on identifying key brain regions involved in this process. It further explored how brain aging affects the regulatory plasticity of brain networks, thereby elucidating the neural mechanisms underlying balance impairments in older adults with MCI from a neuromodulation perspective. The findings provide a theoretical basis for developing evidence-based strategies for fall prevention and intervention.

## 2 Subjects and methods

### 2.1 Study population

#### 2.1.1 Sample size calculation

The required sample size was estimated using G*Power 3.1 software. Based on the findings of Marusic et al. (2019) on prefrontal cortex oxyhemoglobin (HbO) activation during a postural control task (effect size = 0.248), with *α* set at 0.05 and statistical power (1–*β*) at 0.95, the calculations indicated that at least 56 participants were required, with 28 in each group. Considering an anticipated attrition rate of approximately 25%, the sample size was increased to 70 participants, with 35 allocated to each group.

#### 2.1.2 Participants

Seventy participants were initially recruited through community visits and public announcements in neighborhoods near Shandong Sport University, including 35 older adults with MCI and 35 cognitively normal (CN) older adults. Two participants were later excluded—one for not meeting the inclusion criteria and one who voluntarily withdrew during the experiment—resulting in a final sample of 68 participants (34 in each group). The basic characteristics of the participants are presented in Table 1.

**Table 1:** Basic characteristics of participants.

Characteristic	MCI group (*n* = 34)	CN group (*n* = 34)	*t*/*χ*^2^	*p*
Male/female	12/22	6/28	2.720	0.099
Age (years)	67.26 ± 2.77	66.82 ± 3.30	0.595	0.554
MoCA score	21.71 ± 2.22	27.15 ± 1.12	−9.623	<0.001
Height (cm)	163.00 ± 7.69	160.03 ± 6.25	1.748	0.085
Weight (kg)	68.05 ± 9.25	64.75 ± 7.59	1.608	0.113

MCI, mild cognitive impairment; CN, cognitively normal; MoCA, Montreal Cognitive Assessment.

All participants met the following inclusion criteria: (1) age over 65 years; (2) no cardiovascular, respiratory, musculoskeletal, or neurological disorders, and no diseases affecting vision, with normal or corrected-to-normal visual acuity; (3) no use of medications affecting balance or the nervous system within the previous 6 months; (4) no falls within the past 6 months; (5) ability to walk independently; and (6) right-handedness. Additionally, participants with MCI were required to report subjective memory decline, have a Clinical Dementia Rating score of ≥0.5, and a Montreal Cognitive Assessment score between 21 and 25.

This study adhered to the principles of the Declaration of Helsinki and was approved by the Ethics Committee of Shandong Sport University (No. 2024066). All participants were fully informed about the experimental procedures, details, and requirements and provided written informed consent.

#### 2.1.3 Randomization and blinding

Participant grouping was determined according to clinical diagnosis. Because the visual task conditions were directly perceptible to participants, random group assignment and double-blind procedures could not be implemented. To minimize potential bias, a Latin square randomization design was applied within participants to balance the presentation order of visual conditions, thereby controlling for sequence and fatigue effects. All clinical scale assessments and behavioral data collection were conducted by independent evaluators blinded to participants’ diagnostic information. Both center of pressure (COP) and fNIRS data were anonymized, coded, and subsequently processed and analyzed by investigators who were unaware of group assignments.

### 2.2 Experiment design

**Figure 1:**
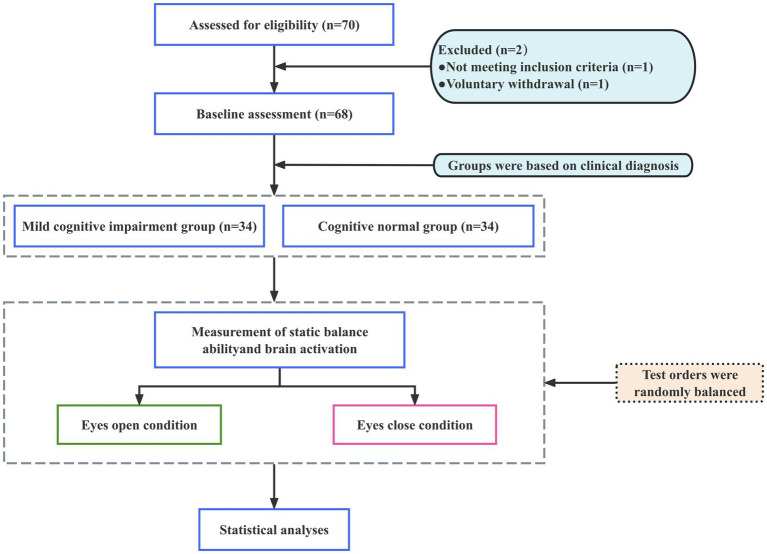
Schematic diagram of study design.

This study employed a 2 (group: MCI, CN) × 2 (visual condition: eyes open, eyes closed) observational controlled design (Figure 1). All participants performed bipedal standing balance tasks under both eyes-open and eyes-closed conditions on a three-dimensional force platform, while fNIRS was used to monitor changes in HbO within regions of interest (ROIs).

#### 2.2.1 Balance testing

**Figure 2:**
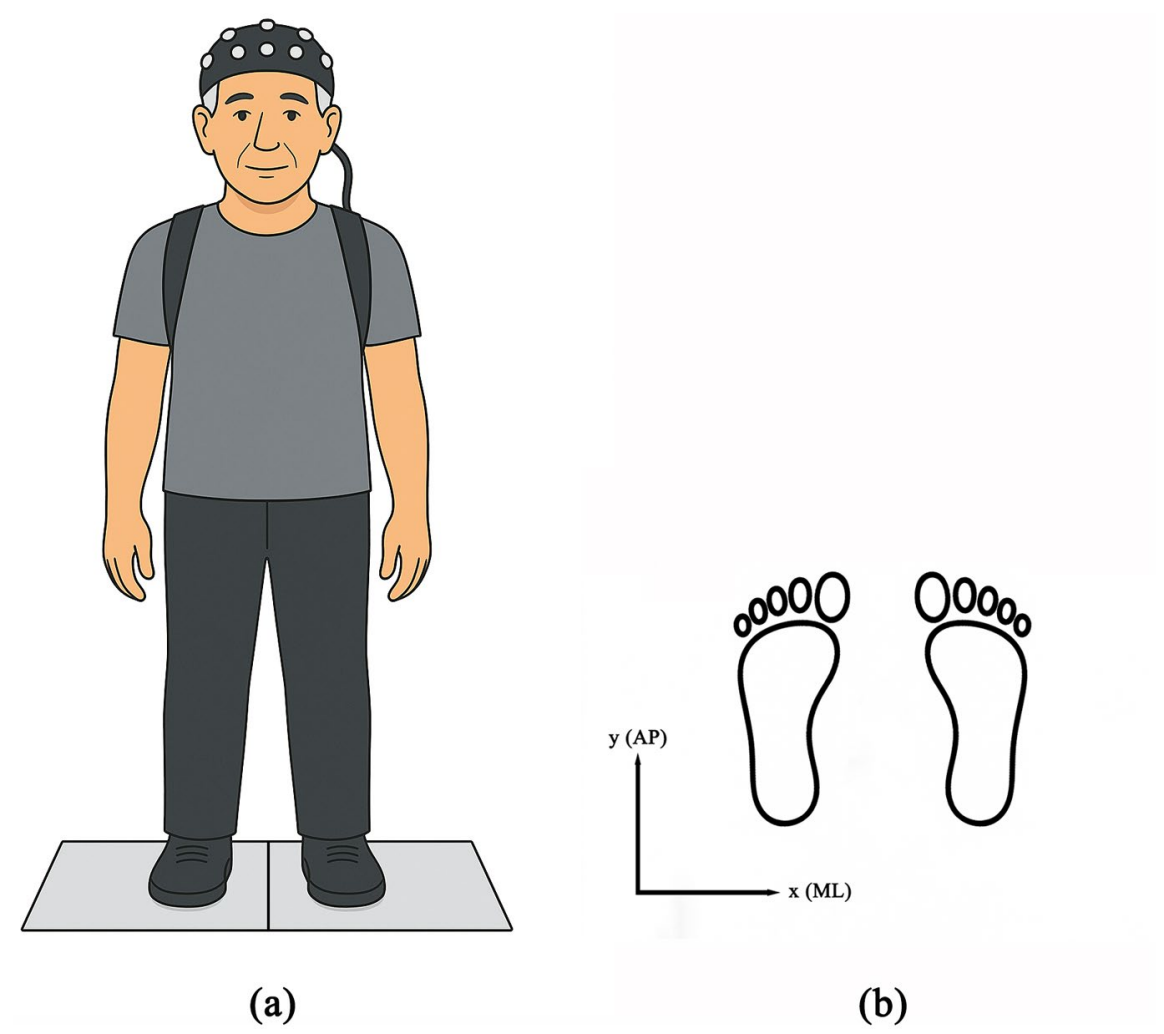
Schematic diagram of the standing task: **(a)** bipedal standing; **(b)** force platform coordinate system.

Participants completed bipedal standing tasks under two visual conditions (Figure 2a): eyes open and eyes closed. Task order was randomized using a Latin square design. Each condition was repeated three consecutive times, with each trial lasting 30 s and a 1-min interval between trials. The COP outcomes for each condition were then averaged across the three trials and used for subsequent statistical analyses. Prior to testing, the force platform was calibrated, with the medio-lateral (ML) direction defined as the x-axis and the anterior–posterior (AP) direction as the y-axis (Figure 2b). Participants then stood barefoot with feet parallel and approximately 20 cm apart, and arms hanging naturally at their sides. During the eyes-open condition, participants were instructed to fixate on a visual reference marker directly ahead. Any leg movement during the trial resulted in the trial being considered invalid. Ground reaction forces were recorded using a three-dimensional force platform (AMTI-BP600900, United States) during standing. The platform measured 90 cm × 60 cm × 10 cm, with a sampling frequency of 1,000 Hz.

#### 2.2.2 fNIRS data acquisition

**Figure 3:**
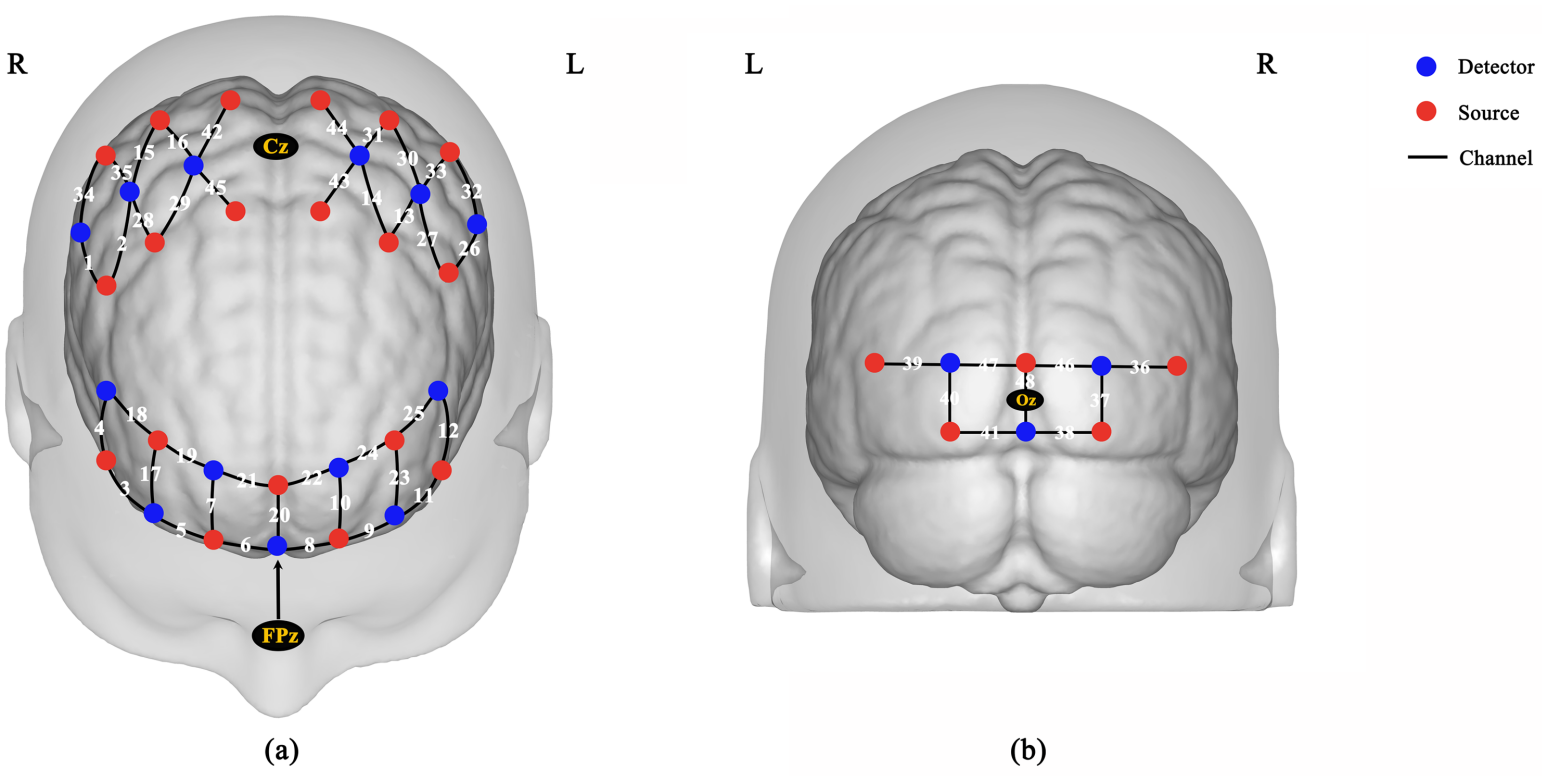
Schematic diagram of optode arrangement on the fNIRS cap: **(a)** top view; **(b)** rear view.

During the standing task, cerebral HbO concentration was measured using a portable near-infrared brain imaging device (NirSmart-3000A, China). The device comprised 24 near-infrared light sources (light-emitting diodes) and 16 detectors (avalanche photodiodes), with light source wavelengths of 730 nm and 850 nm and a sampling rate of 11 Hz. Based on prior research, this study designated the frontal, parietal (Xu et al., 2024; Gozdas et al., 2024), and occipital lobes (Fritz et al., 2019) as regions of interest (Yuan et al., 2015). Following the international 10–20 electroencephalography system, 48 effective channels were configured with emitters and detectors arranged in alternating patterns. The mean distance between adjacent light electrodes was 3 cm (range: 2.7–3.3 cm, Figure 3).

Additionally, channel coordinates were acquired using a 3D positioning device (NirSpace, China) and mapped to corresponding brain regions based on Brodmann Areas using Chris Rorden’s MRIcro (Table 2).

**Table 2:** Corresponding brain regions for each channel.

Hemisphere	Brain region	Channels
Left	Prefrontal cortex	8, 9, 10, 11, 12, 20, 22, 23, 24, 25
	Primary somatosensory cortex	30, 32, 33
	Primary motor cortex	31, 44
	Premotor and supplementary motor cortex	13, 14, 27, 43, 26
	Visual cortex	39, 40, 41, 47, 48
Right	Prefrontal cortex	3, 17, 19, 4, 18, 5, 7, 21, 6
	Primary somatosensory cortex	34, 15, 35
	Primary motor cortex	16, 42
	Premotor and supplementary motor cortex	1, 2, 28, 29, 45
	Visual cortex	36, 37, 38, 46

### 2.3 Primary observation indicators

#### 2.3.1 Balance indicators

The primary balance indicator selected was the COP. COP data were smoothed using a Butterworth low-pass filter with a cutoff frequency of 10 Hz (Hijmans et al., 2009). Subsequently, the following parameters were calculated from the COP data: root mean square displacement (RMS), ML-RMS, AP-RMS, mean velocity (V), ML-V, AP-V, and sway area (AREA) (Kodesh et al., 2021).

#### 2.3.2 Brain network feature analysis

(1) Functional connectivity analysis of brain networks

To quantify the strength of functional connectivity between brain regions, HbO time series were extracted from each ROI. When an ROI included multiple measurement channels, a single ROI-level HbO time series was generated by calculating the arithmetic mean of the HbO time series across all channels assigned to that ROI. Pearson correlation coefficients (*r*) were calculated between all ROI pairs to construct a correlation matrix reflecting temporal synchrony. Negative correlations were set to 0, and all *r* values were Fisher z-transformed before network analysis to improve normality.

(2) Graph-theory-based brain network computation

Graph-theoretical analysis was performed using the GRETNA toolbox. To ensure network connectivity across all participants, the minimum sparsity threshold (S = 0.2667) was determined using the Gretna_get_rmax function (Watts and Strogatz, 1998). The upper sparsity bound was set to 0.50 to avoid excessive network density (Duan et al., 2012; Tian et al., 2011). Binary undirected networks were constructed using proportional thresholding across a sparsity range of 0.2667–0.50 (step = 0.01). For each threshold, global efficiency (Eg), local efficiency (Eloc), small-worldness (*σ*), and nodal efficiency (Ne) were computed. Small-world parameters were normalized against 1,000 degree-preserving random networks generated by rewiring while preserving node degree. For each metric, the area under the curve (AUC) was calculated as the sum across thresholds multiplied by the sparsity increment (Δsparsity = 0.01). All reported results represent these AUC values across the full sparsity range.

### 2.4 Data processing and statistical analysis

#### 2.4.1 fNIRS data processing

fNIRS data were preprocessed using Homer2 software based on Matlab (R2021a). First, raw optical intensity signals were converted to optical density. Subsequently, motion artifacts exhibiting signal changes exceeding 10% standard deviation within 0.5 s were removed. A bandpass filter (0.01–0.1 Hz) was applied to eliminate common noise sources, including respiration, heartbeat, and Mayer waves (Maidan et al., 2016). Finally, optical density signals were converted to hemoglobin concentration changes using the modified Beer–Lambert law, and global trends were normalized through mean subtraction.

#### 2.4.2 Statistical analysis

All statistical analyses were performed using SPSS 26.0 (IBM, Armonk, NY, United States). Data normality was assessed using the Shapiro–Wilk test, and homogeneity of variance was evaluated with Levene’s test. Between-group differences in demographic and clinical characteristics were examined using independent-samples *t*-tests for continuous variables and chi-square tests for categorical variables. Balance performance (COP) and graph-theoretical metrics (AUC) were analyzed using a 2 (group) × 2 (visual condition) mixed-design analysis of variance (ANOVA), with group as the between-subjects factor and visual condition as the within-subjects factor. For graph-theoretical metrics, multiple comparisons were controlled separately for global and nodal measures. Bonferroni correction was applied across the global metrics to control the family-wise error rate. For the nodal metrics, FDR correction was applied to control for multiple regional comparisons. When significant interaction effects were observed, simple effects analyses were conducted to further examine between-group differences within each visual condition and within-group differences across visual conditions. Effect sizes were reported as partial eta squared (*η*^2^_p_), with values <0.06 interpreted as small, 0.06–0.14 as medium, and ≥0.14 as large effects. All tests were two-tailed, and statistical significance was set at *p* < 0.05, with corrected thresholds applied where appropriate.

## 3 Results

### 3.1 Changes in COP during standing balance

**Figure 4:**
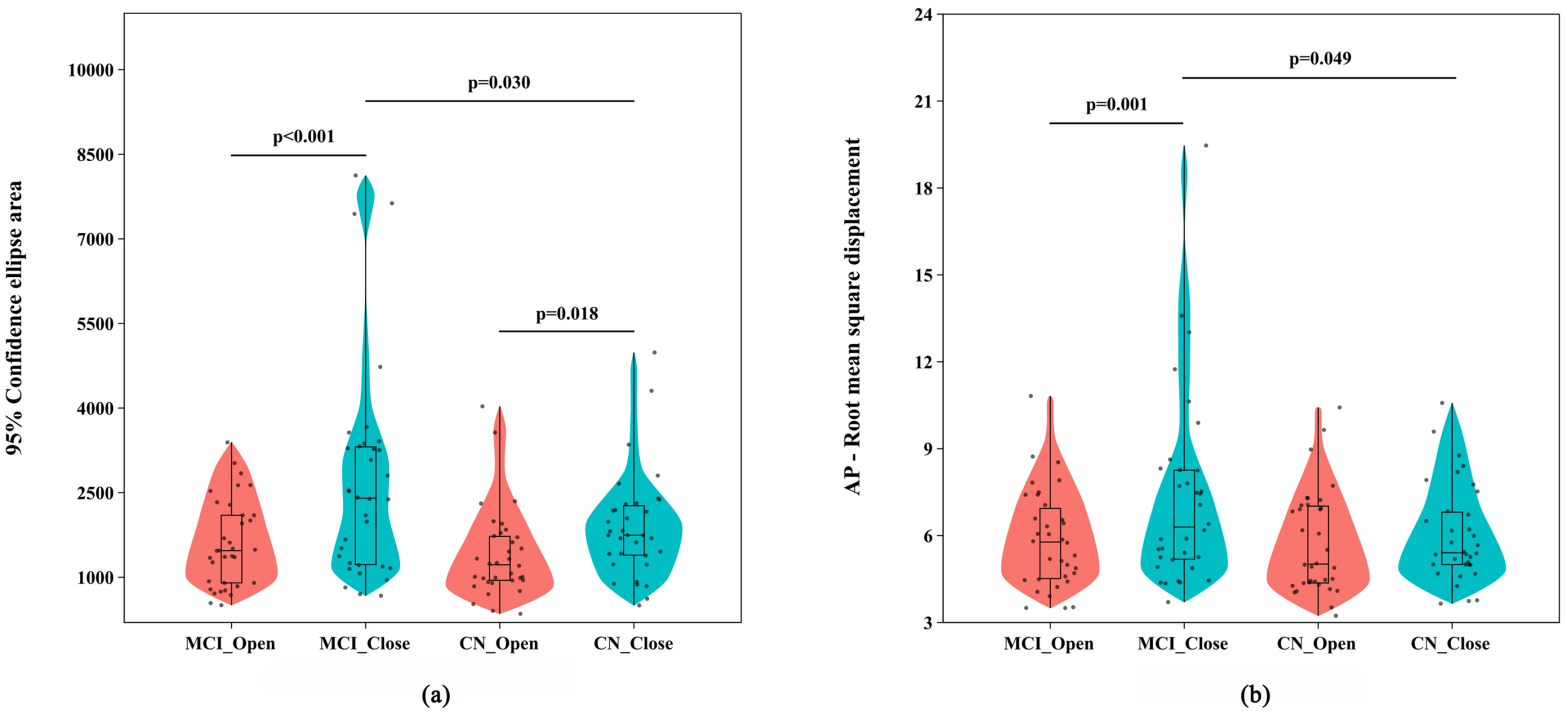
Group comparisons of COP parameters during standing tasks under different visual conditions: **(a)** sway area and **(b)** anterior–posterior root mean square displacement. MCI and CN represent the groups; Open and Close represent the eyes-open and eyes-closed conditions.

A mixed-design ANOVA of COP parameters under different visual conditions (see Table 3) revealed a significant main effect of visual condition for the AREA [*F*_(1,66)_ = 30.294, *p* < 0.001, *η^2^_p_* = 0.315], as well as a significant interaction effect [*F*_(1,66)_ = 4.313, *p* = 0.042, *η^2^_p_* = 0.061]. Similarly, AP-RMS exhibited a significant main effect of visual condition [*F*_(1, 66)_ = 8.627, *p* = 0.005, *η^2^_p_* = 0.116] and a significant interaction effect [*F*_(1,66)_ = 4.157, *p* = 0.045, *η^2^_p_* = 0.059]. ML-V [*F*_(1, 66)_ = 6.166, *p* = 0.016, *η^2^_p_* = 0.085], AP-V [*F*_(1,66)_ = 16.395, *p* < 0.001, *η^2^_p_* = 0.199], and ML-RMS [*F*_(1,66)_ = 14.439, *p* < 0.001, *η^2^_p_* = 0.179] all demonstrated significant main effects of visual condition, but no significant interaction or group effects. Simple effect analyses (see Figure 4) further indicated that, under the eyes-closed condition, AREA (*p* = 0.030) in the MCI group was significantly higher than those in the CN group. Within the MCI group, AREA (*p* < 0.001) and AP-RMS (*p* = 0.001) were significantly higher under the eyes-closed condition than under the eyes-open condition. In the CN group, AREA was also significantly higher in the eyes-closed condition compared with the eyes-open condition (*p* = 0.018).

**Table 3:** Mixed-design ANOVA results of COP parameters under different visual conditions.

Indicator	Condition	Group		Condition effect		Group effect		Interaction effect	
		MCI	CN	*F*	*p*	*F*	*p*	*F*	*p*
AREA	Open	1590.60 ± 778.85	1397.75 ± 790.30	30.294	<0.001	3.975	0.05	4.313	0.042
	Close	2706.75 ± 1896.04	1902.36 ± 949.06						
ML-V	Open	5.52 ± 1.98	5.28 ± 2.34	6.166	0.016	1.263	0.265	0.981	0.326
	Close	6.75 ± 3.74	5.81 ± 1.90						
AP-V	Open	9.8 ± 2.32	9.49 ± 2.58	16.395	<0.001	3.416	0.069	3.682	0.059
	Close	14.01 ± 8.10	10.99 ± 3.36						
V	Open	12.17 ± 2.72	12.62 ± 5.98	15.569	<0.001	0.367	0.547	2.363	0.129
	Close	16.75 ± 9.33	14.63 ± 6.74						
ML-RMS	Open	3.29 ± 1.19	3.49 ± 1.46	14.439	<0.001	3.199	0.078	1.962	0.166
	Close	3.73 ± 1.48	4.19 ± 1.94						
AP-RMS	Open	5.77 ± 1.80	5.82 ± 1.74	8.627	0.005	2.315	0.133	4.157	0.045
	Close	6.68 ± 2.68	6.03 ± 1.69						
RMS	Open	6.95 ± 2.30	7.06 ± 2.25	11.864	0.001	1.235	0.27	1.889	0.174
	Close	7.56 ± 2.33	8.08 ± 3.03						

AREA, the sway area; V, the mean velocity; RMS, root mean square displacement; AP, the anteroposterior direction; ML, the mediolateral direction.

### 3.2 Comparison of brain network graph-theoretical metrics

**Figure 5:**
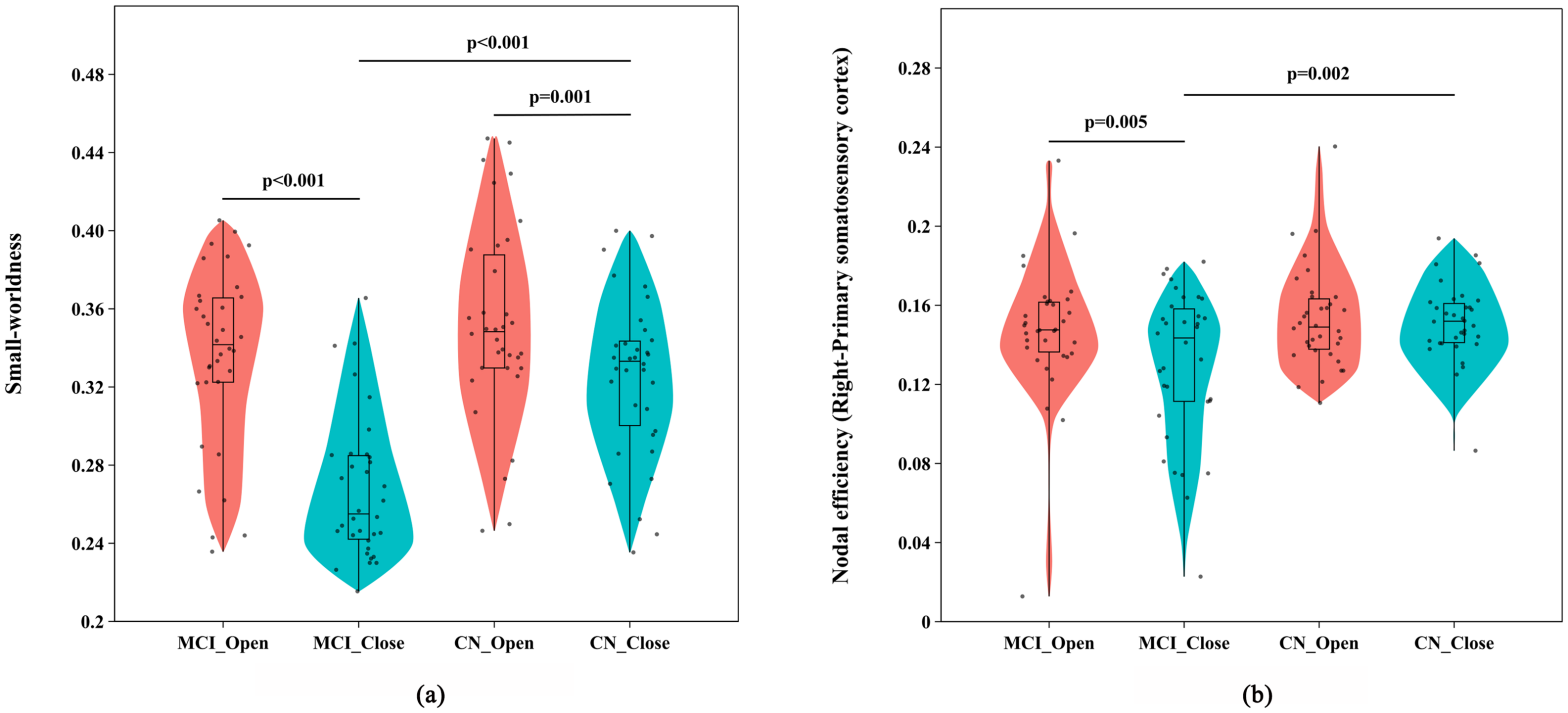
Comparison of graph-theoretical metrics between the two groups during standing under different visual conditions: **(a)** small-worldness and **(b)** nodal efficiency of the right primary somatosensory cortex.

**Figure 6:**
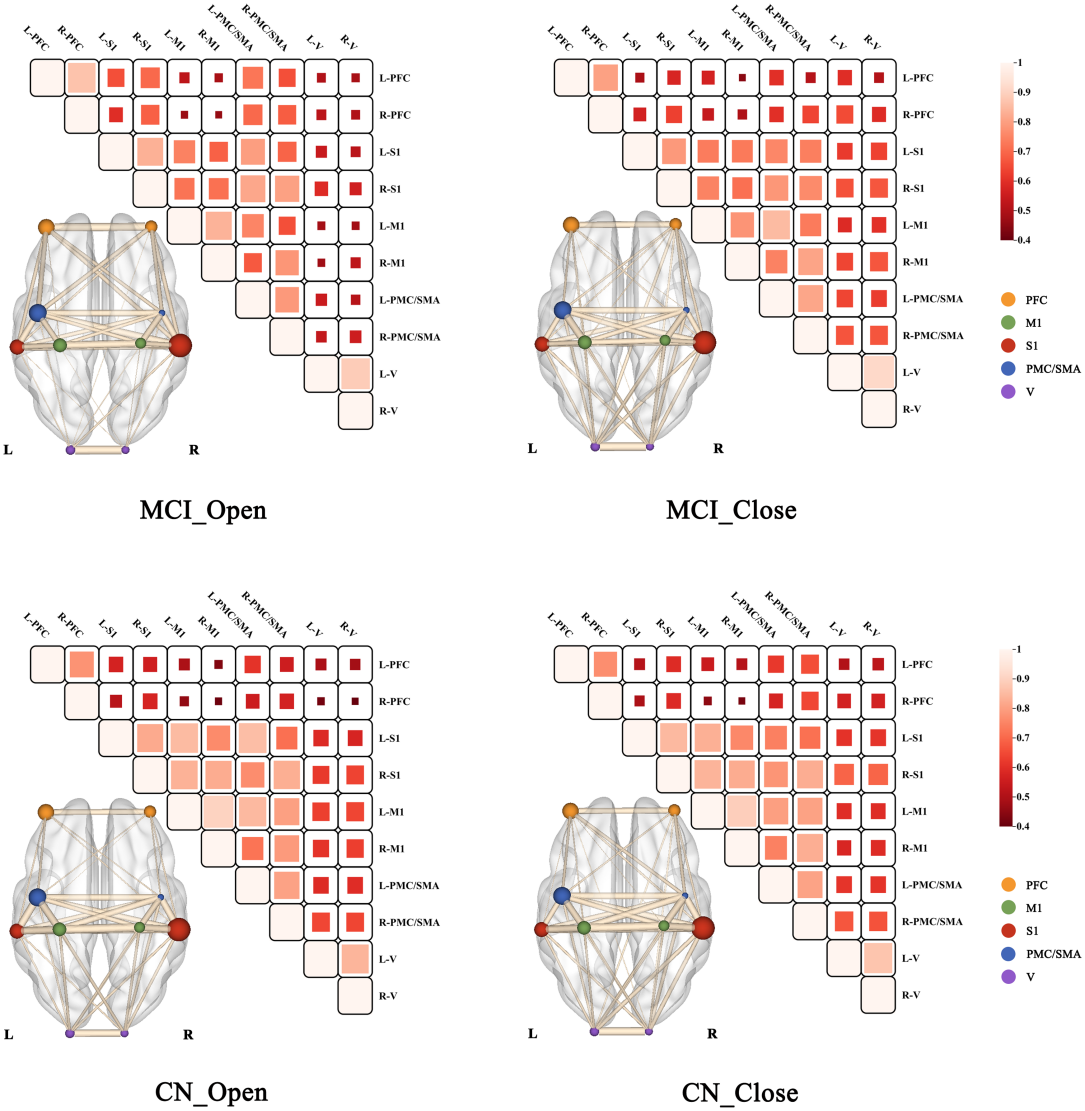
Functional connectivity strength maps during standing under eyes-open and eyes-close conditions in the MCI and CN groups. Thicker connections between ROIs represent higher correlation coefficients (*r* values).

A mixed-design ANOVA (see Table 4) revealed that the *σ* of the brain network showed significant main effects of visual condition [*F*_(1,66)_ = 70.120, *p* < 0.001, *η^2^_p_* = 0.515] and group [*F*_(1, 66)_ = 17.532, *p* < 0.001, *η^2^_p_* = 0.210], as well as a significant interaction effect [*F*_(1,66)_ = 13.400, *p* = 0.001, *η^2^_p_* = 0.169]. In addition, Ne of the right primary somatosensory cortex (R-S1) exhibited significant main effects of visual condition [*F*_(1,66)_ = 5.851, *p* = 0.018, *η^2^_p_* = 0.081] and group [*F*_(1,66)_ = 4.641, *p* = 0.035, *η^2^_p_* = 0.066], as well as a significant interaction effect [*F*_(1,66)_ = 4.244, *p* = 0.043, *η^2^_p_* = 0.060]. Simple effect analyses (see Figure 5) further indicated that, under the eyes-closed condition, both *σ* (*p* < 0.001) and Ne (*p* = 0.005) in the MCI group were significantly lower than those in the CN group. Within the MCI group, *σ* (*p* < 0.001) and Ne (*p* = 0.002) were significantly lower under the eyes-closed condition than under the eyes-open condition. These findings suggest that, under visual deprivation, both global network integration and nodal information transmission efficiency are reduced in older adults with MCI (see Figure 6).

**Table 4:** Mixed-design ANOVA results of brain network parameters under different visual conditions.

Indicator		Condition	Group		Condition effect		Group effect		Interaction effect	
			MCI	CN	*F*	*p*	*F*	*p*	*F*	*p*
*σ*		Open	0.34 ± 0.05	0.35 ± 0.05	70.120	<0.001	17.532	<0.001	13.400	0.001
		Close	0.27 ± 0.04	0.33 ± 0.04						
Eg		Open	0.12 ± 0.01	0.13 ± 0.02	1.372	0.246	0.645	0.425	0.281	0.598
		Close	0.12 ± 0.01	0.12 ± 0.01						
Eloc		Open	0.16 ± 0.02	0.15 ± 0.02	0.146	0.704	0.645	0.425	1.623	0.207
		Close	0.15 ± 0.02	0.16 ± 0.01						
Ne	L_PFC	Open	0.17 ± 0.19	0.21 ± 0.22	2.220	0.141	0.025	0.875	1.904	0.173
		Close	0.25 ± 0.27	0.21 ± 0.2						
	R_PFC	Open	0.19 ± 0.22	0.2 ± 0.22	0.004	0.950	0.025	0.875	0.365	0.548
		Close	0.21 ± 0.19	0.19 ± 0.17						
	L_S1	Open	0.17 ± 0.11	0.16 ± 0.08	0.030	0.862	0.016	0.899	1.768	0.188
		Close	0.15 ± 0.08	0.16 ± 0.12						
	R_S1	Open	0.15 ± 0.03	0.15 ± 0.03	5.851	0.018	4.641	0.035	4.244	0.043
		Close	0.15 ± 0.02	0.14 ± 0.03						
	L_M1	Open	0.16 ± 0.03	0.19 ± 0.15	2.912	0.093	2.254	0.138	0.690	0.409
		Close	0.14 ± 0.03	0.16 ± 0.1						
	R_M1	Open	0.15 ± 0.05	0.15 ± 0.07	0.152	0.698	0.677	0.414	0.302	0.585
		Close	0.15 ± 0.06	0.14 ± 0.05						
	L_PMC	Open	0.17 ± 0.14	0.17 ± 0.12	<0.001	0.994	1.321	0.255	2.878	0.095
		Close	0.14 ± 0.03	0.17 ± 0.12						
	R_PMC	Open	0.18 ± 0.13	0.18 ± 0.15	1.287	0.261	0.014	0.907	0.017	0.897
		Close	0.16 ± 0.02	0.16 ± 0.06						
	L_V	Open	0.24 ± 0.26	0.21 ± 0.22	0.636	0.428	0.963	0.330	0.110	0.741
		Close	0.26 ± 0.27	0.24 ± 0.25						
	R_V	Open	0.2 ± 0.21	0.2 ± 0.19	0.503	0.481	0.192	0.663	0.032	0.859
		Close	0.23 ± 0.25	0.22 ± 0.22						

*σ*, small-worldness; Eg, global efficiency; Eloc, local efficiency; Ne, nodal efficiency; PFC, prefrontal cortex; S1, primary somatosensory cortex; M1, primary motor cortex; PMC, premotor and supplementary motor cortex; V, visual cortex; L and R, left and right hemispheres.

### 3.3 Correlations between COP metrics and brain network characteristics

**Figure 7:**
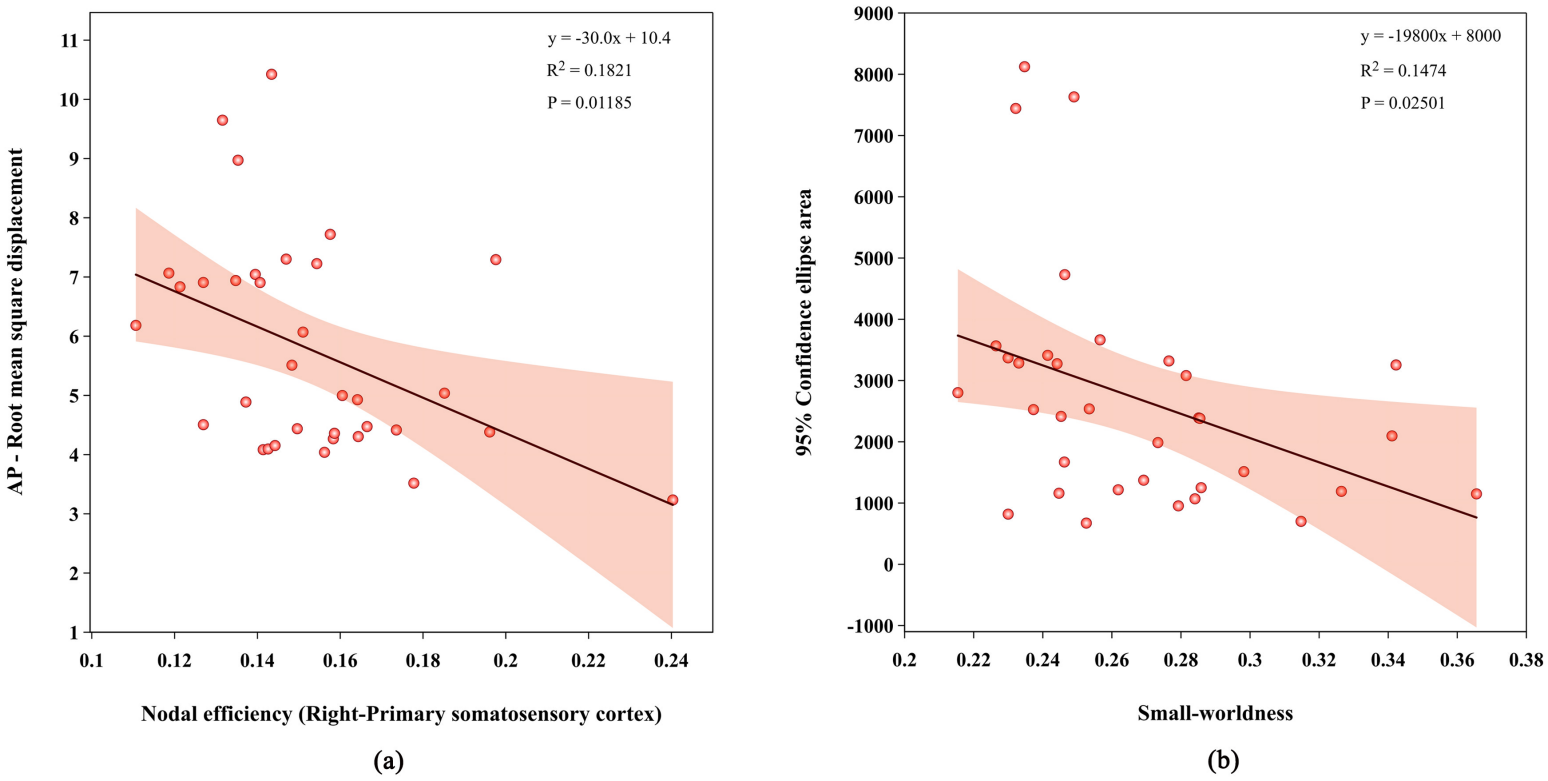
Correlations between COP metrics and brain network characteristics during standing balance: **(a)** correlation between AP-RMS and N_e_ in R-S1 in the CN group under the eyes-open condition; **(b)** correlation between AREA and *σ* in the MCI group under the eyes-closed condition.

Under the eyes-open condition, the AP-RMS in the CN group was significantly negatively correlated with the Ne of R-S1 (*r* = −0.427, *p* = 0.012). Under the eyes-closed condition, the AREA in the MCI group was significantly negatively correlated with *σ* (*r* = −0.384, *p* = 0.025). No significant correlations were found between other COP metrics and brain network characteristics (Table 5; Figure 7).

**Table 5:** Correlations between COP parameters and brain network metrics.

Indicator	Group	Eyes-open				Eyes-close			
		*σ*		Ne (R_S1)		*σ*		Ne (R_S1)	
		*r*	*p*	*r*	*p*	*r*	*p*	*r*	*p*
AREA	MCI	0.066	0.711	−0.267	0.127	−0.384	0.025	−0.024	0.891
	CN	−0.129	0.466	0.321	0.064	0.024	0.894	0.223	0.204
AP_RMS	MCI	0.1	0.574	−0.108	0.544	0.3	0.085	−0.002	0.99
	CN	−0.116	0.513	−0.427	0.012	−0.16	0.365	0.087	0.624

*σ*, small-worldness; Eg, global efficiency; Eloc, local efficiency; Ne, nodal efficiency; PFC, prefrontal cortex; S1, primary somatosensory cortex; M1, primary motor cortex; PMC, premotor and supplementary motor cortex; V, visual cortex; L and R, left and right hemispheres.

## 4 Discussion

This study compared static balance parameters and neural brain network characteristics between elderly individuals with MCI and CN controls under different visual conditions, revealing the influence of visual feedback on balance control ability and its underlying neural mechanisms. The findings showed that during balance maintenance, older adults with MCI exhibited not only a decline in motor control ability but also reduced brain network integration and information transmission efficiency. Furthermore, visual input played a key compensatory and regulatory role in maintaining postural stability.

Behavioral results showed that visual deprivation exacerbated postural instability, and individuals with MCI exhibited a stronger dependence on visual information (Dinse, 2006; Saftari and Kwon, 2018). It is noteworthy that older adults rely on multisensory information to accurately perform daily activities. However, age-related declines in proprioceptive and vestibular functions are common in the elderly (Löffler et al., 2024), and the multisensory integration ability of individuals with MCI is further impaired (Janc et al., 2021). Consequently, when visual information is unavailable, older adults with MCI cannot effectively utilize alternative sensory cues to maintain postural balance (Koen et al., 2019; Kucharik et al., 2020). Furthermore, postural regulation and feedback adjustment under eyes-closed conditions require greater cognitive resource allocation. However, older adults with MCI possess limited cognitive capacities—particularly in attention, executive function, and spatial cognition—which may further increase body sway during balance maintenance (Hwang and Lee, 2017).

From the perspective of neural regulatory mechanisms, the abnormal alterations in brain network topology observed in elderly individuals with MCI under different visual conditions suggest functional impairments in sensory integration and neural network modulation. The absence of visual input generally reduces the organizational efficiency of brain networks; however, individuals with MCI exhibit greater network vulnerability and diminished regulatory capacity (Jiang et al., 2022). Previous studies have shown that a reduction in *σ* indicates a transition of functional connectivity from an efficient “small-world” configuration to a more randomized network, disrupting the balance between local clustering and global integration, and thereby impairing the efficiency of neural information transmission (Humphries et al., 2006). The primary somatosensory cortex (S1), as a key node for processing somatosensory input during postural control, plays a crucial role in perceiving body position and integrating information from proprioceptive and tactile inputs (Wang et al., 2025a). A decline in its nodal efficiency indicates reduced capacity for information transmission and integration within the brain network, making it difficult to promptly integrate postural feedback signals. This reduction also weakens information exchange between the S1 and somatosensory integration regions—such as the premotor cortex, parietal lobe, and occipital lobe—thereby limiting fine postural adjustments and diminishing overall postural stability (Barollo et al., 2022). Therefore, when visual cues are deprived, CN older adults can maintain postural stability by strengthening functional connectivity within somatosensory and vestibular pathways. In contrast, individuals with MCI, due to reduced cortical plasticity and impaired cross-modal integration, fail to achieve effective compensatory activation, ultimately resulting in a significant decline in network integration efficiency (Jiang et al., 2022; Leng et al., 2023). These findings confirm the neural modulatory role of visual input in maintaining postural control. Moreover, the greater tendency toward network randomization and reduced sensorimotor feedback efficiency in MCI individuals under visual deprivation further reflect the vulnerability of their sensory integration system. Such network vulnerability at the neural level may constitute an important mechanistic basis for postural control impairments in elderly individuals with MCI.

Further analysis of the correlations between balance performance indicators and brain network topological characteristics revealed that when visual cues are fully available, balance control primarily relies on the integrative processing of visual and somatosensory information (Dalton et al., 2017). This finding is consistent with previous studies suggesting that the somatosensory cortex plays a central role in maintaining static postural stability. Particularly under visually dominant conditions, increased activation of the somatosensory cortex enhances the precision of sensory feedback and the temporal efficiency of motor control (Song et al., 2016; Baradaran et al., 2025). In contrast, when visual input is limited, individuals with MCI rely on functional cooperation across multiple brain regions to compensate for the loss of visual information. They attempt to maintain postural stability by enhancing large-scale information integration and efficient neural transmission throughout the brain (Xu et al., 2024). However, the overall lower small-world properties observed in the MCI group indicate reduced efficiency in both network integration and segregation. Such network inefficiency may constrain the flexibility of sensory and postural regulation, ultimately leading to impaired balance performance (Jie et al., 2014; Jiang et al., 2022). Notably, the correlation findings reveal the network-level basis of postural control from the perspective of neural mechanisms. Balance regulation depends not only on the integration of external sensory inputs but also on the structural coordination and efficiency of information transmission within the brain network.

In summary, this study revealed the coupling mechanism between brain network topological structure and postural behavioral characteristics in elderly individuals with MCI during balance control, emphasizing the crucial regulatory role of visual input in maintaining postural stability. The findings indicate that the decline in balance ability arises not only from degeneration of peripheral sensory and motor systems but is also closely associated with reduced efficiency and weakened information integration within central neural networks. Based on the theory of brain plasticity (Koch and Spampinato, 2022), this study provides a neurophysiological foundation for developing task-specific balance training programs that incorporate visual input modulation and brain network characteristics. Future studies may further integrate non-invasive brain stimulation techniques (e.g., tDCS, TMS) to modulate the excitability of key brain regions and implement balance training under multi-sensory integration conditions. Such approaches are expected to enhance brain network efficiency and sensorimotor integration in elderly individuals with MCI, thereby improving balance control and reducing fall risk.

## 5 Conclusion

Visual input significantly affects the balance ability of elderly individuals with MCI, leading to reduced postural stability and lower integration efficiency of brain functional networks during static balance tasks. Compared with the CN group, individuals with MCI exhibited significantly impaired integration of sensory and motor information. Further correlation analysis revealed a negative association between balance metrics and brain network efficiency, suggesting that the integrity of functional neural connectivity plays a crucial role in maintaining postural stability. Overall, individuals with MCI demonstrated increased dependence on visual input, while visual deprivation revealed the vulnerability of their neural network integration capacity. This study elucidates the central mechanisms underlying visual perception in balance control disorders among elderly individuals with MCI, providing a theoretical basis for targeted motor interventions and early functional rehabilitation.

## Data Availability

The original contributions presented in the study are included in the article/supplementary material, further inquiries can be directed to the corresponding author.
